# Cellular and Mitochondrial NAD Homeostasis in Health and Disease

**DOI:** 10.3390/cells12091329

**Published:** 2023-05-06

**Authors:** Jaylyn Waddell, Rehana Khatoon, Tibor Kristian

**Affiliations:** 1 Department of Pediatrics, University of Maryland School of Medicine, Baltimore, MD 21201, USA; jwaddell@som.umaryland.edu; 2 Department of Anesthesiology and the Center for Shock, Trauma and Anesthesiology Research (S.T.A.R.), University of Maryland School of Medicine, Baltimore, MD 21201, USA; rkhatoon@som.umaryland.edu; 3 Veterans Affairs Maryland Health Center System, 10 North Greene Street, Baltimore, MD 21201, USA

**Keywords:** mitochondria, NAD, brain

## Abstract

The mitochondrion has a unique position among other cellular organelles due to its dynamic properties and symbiotic nature, which is reflected in an active exchange of metabolites and cofactors between the rest of the intracellular compartments. The mitochondrial energy metabolism is greatly dependent on nicotinamide adenine dinucleotide (NAD) as a cofactor that is essential for both the activity of respiratory and TCA cycle enzymes. The NAD level is determined by the rate of NAD synthesis, the activity of NAD-consuming enzymes, and the exchange rate between the individual subcellular compartments. In this review, we discuss the NAD synthesis pathways, the NAD degradation enzymes, and NAD subcellular localization, as well as NAD transport mechanisms with a focus on mitochondria. Finally, the effect of the pathologic depletion of mitochondrial NAD pools on mitochondrial proteins’ post-translational modifications and its role in neurodegeneration will be reviewed. Understanding the physiological constraints and mechanisms of NAD maintenance and the exchange between subcellular compartments is critical given NAD’s broad effects and roles in health and disease.

## 1. Introduction

The mitochondrion represents a unique intracellular organelle due to its evolutionary origin, dynamic properties, and essential role in cellular energy metabolism. Furthermore, due to their symbiotic nature mitochondria can also modulate a subcellular organelle’s function by sharing common metabolic intermediates with the rest of the cell compartments. Therefore, any disturbance in mitochondrial homeostasis has a significant impact on cellular function or survival, and is associated with many disease pathologies, particularly in high-energy-demanding tissue such as brain tissue [1,2,3,4].

One of the ubiquitous metabolic intermediates located in most intracellular subcompartments is nicotinamide adenine dinucleotide (NAD). Cellular energy metabolism is strongly reliant on the presence of NAD and its reduced form NADH (or its phosphorylated forms NADP and NADPH), which serve as cofactors for numerous enzymatic reactions. NAD is required not only as a component of reactions that are involved in the reduction or oxidation of intermediate metabolites, but also serves as a substrate for post-translational modifications that alter gene expression and modulate several cellular processes, including lipid and sterol metabolism, the inflammatory and stress response, and aging. The intracellular distribution of NAD is compartmentalized into a discrete cytosolic and subcellular compartment represented mainly by cytosol, nuclei, and mitochondria.

In this review, we will discuss the mechanisms of cellular and mitochondrial NAD homeostasis, as well as the compartmentation of NAD with implications for treatment strategies in pathophysiology for acute brain injury or neurodegenerative diseases.

## 2. NAD Synthesis and Degradation in Mitochondria and Other Cellular Subcompartments

**Figure 1:**
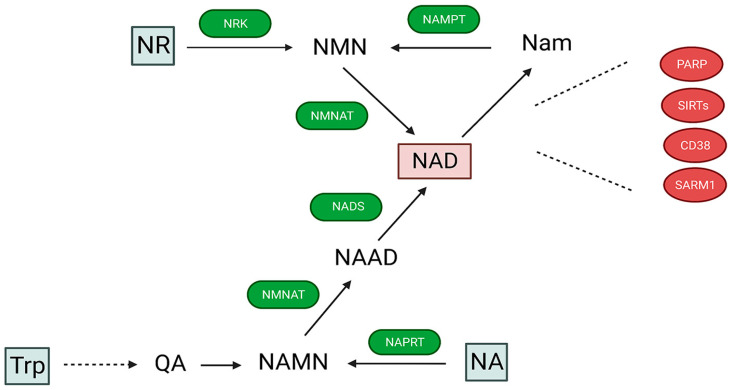
Metabolic pathways of NAD biosynthesis and NAD degradation. The main source of NAD is from the salvage pathway, where it is generated by enzymatic reactions that use nicotinamide (Nam) to generate nicotinamide mononucleotide (NMN) via nicotinamide phosphotransferase (NAMPT) activity. The NMN can also be formed by phosphorylation of nicotinamide riboside (NR) via NR kinase (NRK). NMN is then converted to NAD by nicotinamide mononucleotide adenylyl transferase (NMNAT). In the Preiss–Handler pathway, nicotinic acid adenine dinucleotide (NAMN) is synthesized from nicotinic acid (NA). Subsequently, NAMN is converted by NMNAT into nicotinic acid adenine dinucleotide (NAAD), which is then amidated to NAD by NAD synthetase (NADS). De novo pathway starts from tryptophane (Trp), and also leads to formation of NAMN by conversion from quinolinic acid (QA). NAD is consumed during poly-ADP-ribosylation or acetylation of proteins driven by PARP or sirtuins. Additionally, NAD is used as substrate by CD38 and SARM1 enzymes.

The intracellular NAD levels are affected by the activity of the NAD-synthetizing enzymes, by the rate of NAD consumption by NAD glycohydrolases, and by an exchange or transport of NAD between the individual subcellular compartments. NAD is generated either via de novo synthesis from quinolinic acid, a product of tryptophan catabolism in the kynurenine pathway [5], or via the salvage pathway, where a byproduct of NAD catabolism nicotinamide (Nam) serves as a substrate for the enzyme nicotinamide phosphoribosyl transferase (Nampt), which forms a nicotinamide mononucleotide (NMN) [6] (Figure 1). An NMN can also be generated by phosphorylation of the B3 vitamin from nicotinamide riboside (NR) via the NR kinase (NRK) [7]. The NMN is then used to generate NAD in the presence of ATP by nicotinamide mononucleotide adenylyl transferase (NMNAT) [8]. There are three isozymes of NMNAT (NMNAT1-3), with each localized in different cellular compartments. NMNAT1 is localized in the nucleus, NMNAT3 is in the mitochondria, and NMNAT2 is in the cytosol and Golgi [9]. However, recently it was reported that NMNAT can also function as a chaperone, and it is preferentially linked to the facilitation of protein folding [10,11,12]. Interestingly, recent studies were unable to confirm the NMNAT3 activity in human mitochondria [13,14], and the deletion of NMNAT3 did not affect mitochondrial respiration or mitochondrial NAD levels, calling into question NMNAT3 as an effective NAD-synthetizing enzyme [15]. Additionally, for NMNAT3 to synthetize NAD, the NMN needs to be transported from the cytosol into the mitochondrial matrix. Although an NMN-specific transporter SLC12A8 was recently identified, its intracellular or mitochondrial localization has not been reported [16]. 

NAD is also generated by the Preiss–Handler pathway from nicotinic acid (NA) or nicotinic acid riboside (NAR), which yields to the nicotinic acid mononucleotide (NAMN) and then the nicotinic acid adenine dinucleotide (NAAD), and is finally converted to NAD by NAD synthetase (NADS) (for review, see [17]). 

## 3. Intracellular NAD Degradation Enzymes and Their Compartmentation 

There are two major groups of NAD-consuming enzymes in cells. Both are linked to the post-translation modification of proteins, mainly via deacetylation and ADP-ribosylation. Class III deacetylase sirtuins (Sirts) require NAD for their activity [18]. This is because Sirt-mediated deacetylation is coupled with the cleavage of NAD into Nam and ADP-ribose (ADPR). Then, the acetyl moiety from the target protein is transferred onto ADPR, forming O-acetyl-ADP-ribose. In mammals, seven Sirt homologs were reported (Sirt1-7) [19]. Sirt1, Sirt6, and Sirt7 are nuclear proteins [20]. Sirt2 is localized in the cytosol [21], but can also deacetylase histones [22], suggesting it is able to shuttle between nuclear and cytosolic compartments. Although there are three isoforms of sirtuins located in mitochondria (Sirt3-5), only Sirt3 is a major protein deacetylase [23]. Sirt5 mediates protein desuccinylation [24] and Sirt4 acts as a mono-ADP-ribosyl transferase [25]. 

The NAD-dependent ADP-ribosylation of proteins can be either mono-ADP-ribosylation or poly-ADP-ribosylation with the concomitant release of Nam [2]. Extensive poly-ADP-ribosylation by poly(ADP-ribose) polymerase 1 (PARP1) was suggested as a major mechanism of NAD consumption following oxidative stress induced by brain ischemia or traumatic brain injury [3,26,27,28,29,30]. PARP1 was reported to be localized in the nucleus; however, there are several reports suggesting the presence of intramitochondrial PARP1 (mtPARP1) [31,32,33]. Interestingly, the mtPARP1 Km value for NAD (22 μm) is significantly lower than the Km value of nuclear PARP1 (210 μm) [34,35]. Thus, mitochondria-specific PARP activity appears to be higher than nuclear activity, suggesting that following PARP activation there is a higher NAD turnover in the mitochondrial matrix when compared to the nucleus or cytosol. Since, until recently, in mammalian cells the mitochondrial and cytosolic NAD pools were considered to be isolated [36,37,38], these findings would also suggest that stimulated mtPARP requires more active resynthesis/replenishment of intramitochondrial NAD. 

In addition to its role as a regulator of protein post-translation modification, NAD is a substrate for ADP-ribosyl cyclase CD38 and its homologue CD157, which uses NAD to generate a second messenger cyclic-ADP-ribose (cADPR) that releases calcium from endogenous intracellular stores [39]. CD38 expression was detected in the perikaryal region and dendrites of hippocampal neurons [40], cerebellar Purkinje cells [41], astrocytes [42], and microglia [43]. The higher activity of CD38 and its contribution to brain NAD degradation was observed after ischemic insult [44], and increased levels of CD38 during aging were proposed as one of the major mechanisms of the age-dependent reduction in cellular NAD pools [45]. However, since CD38 is mostly an ectoenzyme with its catalytic site facing toward the extracellular environment [46], it is not clear how it can directly modulate the intracellular NAD levels. One possibility is that CD38 regulates the metabolism of the NAD precursor (NMN or NR) in the extracellular fluids [47]. Furthermore, there are reports suggesting that a small fraction of CD38 can have its catalytic site facing the cytosol. Additionally, CD38 localized in the nuclear or mitochondrial membrane will have access to intracellular NAD, and its activation could lead to the significant consumption of cellular NAD [48,49]. 

Recently, another protein, SARM1 (sterile alpha and Toll/interleukin-1 receptor motif-containing 1), was identified as a major NAD-degrading enzyme that plays a central role in the mechanism underlying axonal degeneration [50,51]. 

Thus, there are several enzymes that use NAD as a substrate and, when overactivated due to pathologic stress or aging, might lead to a significant reduction in cellular NAD levels and bioenergetic failure. However, little is known about which cellular compartment is most affected under specific pathophysiologic conditions and whether there is a possible exchange of NAD between individual intracellular NAD pools to compensate for the insult-induced NAD degradation, or if the replenishment is taking place only via the salvage pathway.

## 4. Mitochondria and Intracellular NAD Exchange 

The differential intracellular distribution of enzymes contributing to NAD replenishment or degradation suggests that each subcellular organelle possesses a specific mechanism for maintaining NAD levels. This notion is supported by the finding that segregated NAD pools have been demonstrated not only in mitochondria, but also in nuclei, peroxisomes, the Golgi apparatus, and the endoplasmic reticulum [52]. Additionally, such compartmentalization of metabolites and small molecules within the cell is not unprecedented since calcium ions [53], ATP [54], and acetyl-CoA [55] have also been reported to be compartmentalized. The NAD exchange mechanisms between the cytosol and nucleus are not clear. However, the diameter of the nuclear pore would not be expected to impose a physical barrier to NAD diffusion between the two compartments [56], as the selective depletion of NMNAT2 or NMNAT1 will limit NAD availability only in the cytosol or only in the nucleus [57].

Different cellular metabolic pathways are compartmentalized and interconnected via transport mechanisms of individual intermediate metabolites. The effective symbiotic function between mitochondria and the rest of the cell is ensured by the presence of mitochondrial carriers (MCs) that shuttle a variety of metabolites across the inner mitochondrial membrane and which are encoded by the solute carrier family (SLC) genes [58,59]. All MCs are proteins encoded in nuclear DNA and must be imported into the inner mitochondrial membrane. These carriers can be electrogenic, thus resulting in the transport of a net positive or negative charge, or they can be electroneutral. For example, the ADP/ATP carrier catalyzes the exchange of ADP^3−^ for ATP^4−^ that results in a charge imbalance. However, the carrier for inorganic phosphate (Pi; carries one to three negative charges and is pH dependent) represents an electroneutral transport since Pi anions are transported together with an equivalent amount of hydrogen cations (H^+^) or are exchanged for hydroxyl anions (OH^−^) [58]. 

The compartmentation of NAD-synthetizing enzymes into individual subcellular compartments led for many years to the assumption that NAD is not transported between the individual intracellular organelles and cytosol and that NAD can be generated in the mitochondrial matrix [37,38,60]. Furthermore, there are several studies that suggested a relatively autonomous and independent mitochondrial NAD pool that differs from the cytosolic one [38,61]. 

### 4.1. NAD and Mitochondrial Inner Membrane Permeability 

Translocation of NAD from the mitochondria was only associated with the mitochondrial permeability transition (MPT) pore opening that is formed in the inner mitochondrial membrane under stress conditions when mitochondria are overloaded with calcium and/or exposed to oxidative stress [62,63]. This large conductance pore is nonspecific for NAD since all solutes of a molecular weight up to 1500 Da can pass from the mitochondrial matrix into the cytosol [64]. The opening of such a large pore leads to the leak of matrix solutes into the cytosol, causing a loss of the mitochondria membrane potential and organelle swelling [64,65,66]. Although the release of NAD via this “mega” channel is relatively slow, taking several minutes, a prolonged activation of the MPT leads to swelling-induced physical disruption of mitochondrial integrity [64,65,66]. The translocation of NAD from the mitochondria to the cytosol due to an MPT opening likely contributes to the pathologic reduction in mitochondrial NAD pools and bioenergetic failure that are observed following ischemic or traumatic insult, contributing to cell death [3,67]. As a neuroprotective approach, one can either inhibit the MPT activation by using MPT inhibitors such as cyclosporine A (CsA) [68,69] or stimulate the post-insult NAD replenishment by administering a substrate for NAD synthesis [3,70,71,72]. 

### 4.2. Cell Membrane NAD Permeability 

Until recently, it was commonly believed that NAD cannot pass through the cellular or organelle membranes in mammalian brain cells. However, several reports have indicated that extracellularly administered NAD can restore the intracellular NAD stores in both astrocytes and neurons [73,74,75]. The intracellular increase in NAD pools after extracellularly applied NAD was prevented by a purinergic receptor P2X7 inhibitor, suggesting that NAD was able to enter the cells via P2X7-gated channels [74]. It has also been reported that connexin 43 channels located in the plasma membrane can allow NAD to enter the intracellular compartment [76]. However, another possibility is that the exogenous NAD is degraded by ectonucleotidases and the generated metabolic intermediates are taken up into the cytosol via P2X7-gated pores [77].

### 4.3. Mitochondria-Specific NAD Transport 

**Figure 2:**
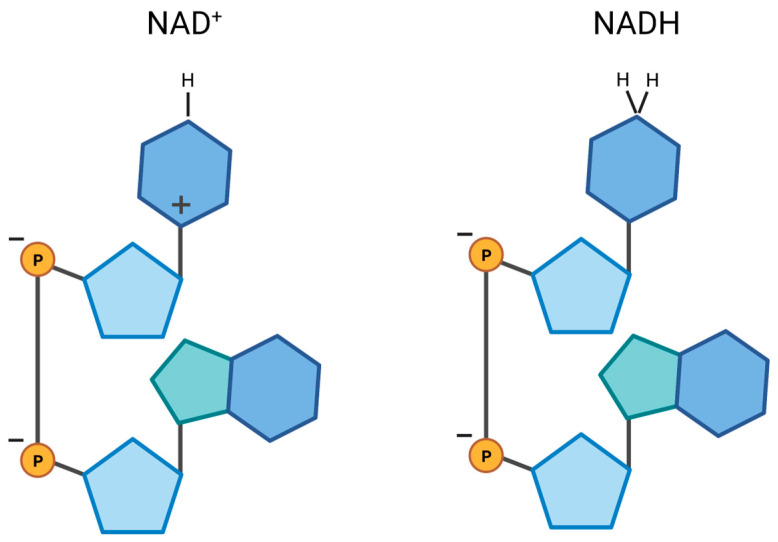
Schematic illustration of NAD and NADH molecules and their charges. Although the abbreviation for nicotinamide adenine dinucleotide is written with a plus sign (NAD^+^), its net charge is negative due to the presence of two phosphate groups. Similarly, NADH has a net charge of negative two.

The oxidized form of nicotinamide adenine dinucleotide is commonly written with a plus sign as NAD^+^, suggesting that it is a cation in a solution. However, since NAD is composed of two ribose molecules that are connected via two phosphate groups that each carry one negative charge, the net charge of NAD is minus one (NADH has two net negative charges; Figure 2). Therefore, translocation of NAD from the cytosol across a mitochondrial membrane with a negative membrane potential inside mitochondria at about −180 mV requires a high driving force to facilitate the transport. This can be represented by the concentration gradient of the solute between the intramitochondrial and cytosolic compartment that is co-transported or exchanged with NAD and the electrochemical potential across the inner membrane generated by the mitochondrial respiratory enzymes. Transport via an NAD-specific channel in the mitochondrial inner membrane is highly unlikely since it would lead to an intramitochondrial NAD concentration three orders of magnitude lower when compared to a cytosolic concentration at the Nernst equilibrium. The mitochondrial NAD levels are higher than the cytosolic levels, with the relative difference being cell-type dependent [61]. In neurons mitochondrial NAD pools are larger, making up about 50% of the total cellular NAD when compared to astrocytes, where they represent about 25% [78]. Therefore, it is more likely that an NAD-specific transporter is contributing to the replenishment of intramitochondrial NAD pools. In plant and yeast mitochondria, an NAD-specific transporter was identified more than a decade ago [79,80]. 

**Figure 3:**
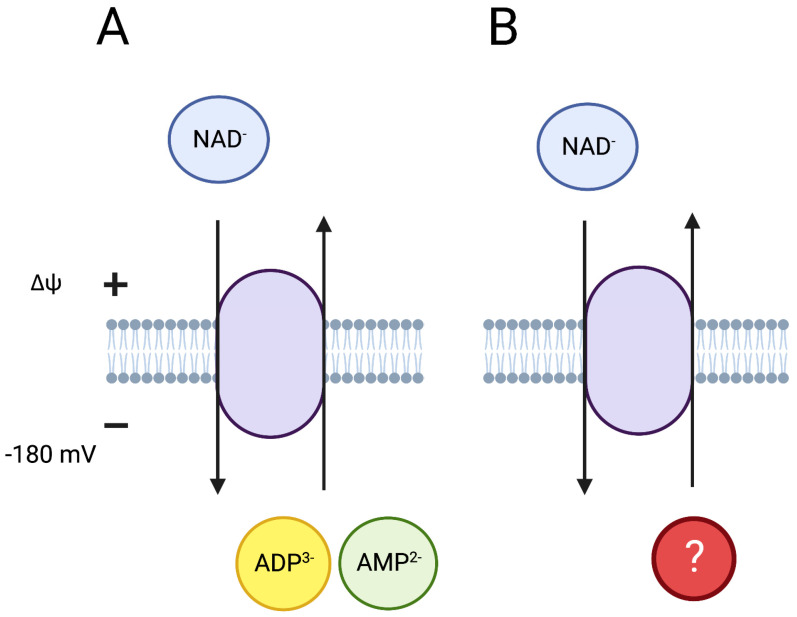
Mitochondrial NAD transporter in plants and yeast (**A**), and in mammalian cells (**B**). In plants and yeast mitochondria NAD is transported from cytosol to the mitochondrial matrix for exchange with ADP or AMP. Mammalian transporter SLC25A51 is specific for NAD; however, it is not known whether it works as a co-transporter and which metabolite is exchanged for NAD. NAD^−^ is shown with one net negative charge (see Figure 2). The exchange for ADP^3−^ or AMP^2−^ in plant and yeast mitochondria is driven by their concentration gradient and the mitochondrial membrane potential (ΔΨ).

Recently, the mitochondrial carrier SCL25A51 that is specific to NAD was also reported in mammalian mitochondria by several laboratories [15,81,82]. SLC25A51 shows specificity to NAD when compared to other metabolites or the NAD precursor NMN [15,81,82]. However, reports regarding the NADH transport ability are conflicting. The NADH transport by this carrier is less likely since NADH has one extra negative net charge when compared to NAD, thus requiring even more energy for transport across the inner mitochondrial membrane if the transporter is not electroneutral. Mitochondrial NAD carriers in plant mitochondria counter-exchange ADP^3−^ or AMP^2−^ for NAD, thus giving a net positive charge to the exchange (three or two negative charges transported out and one negative charge with NAD transported into the mitochondrial matrix). Thus, the mitochondrial NAD uptake is driven by the negative membrane potential and the concentration gradient of ADP/AMP across the inner mitochondrial membrane (Figure 3). However, it is not known whether the mammalian mitochondrial carrier SCL25A51 is a co-transporter or an exchanger, and which metabolites are synergistically transported with NAD. The replenishment of depleted mitochondrial NAD pools by supplying external NAD is slow and takes several minutes [15,81,82]. Therefore, to determine the effects of the transporter on the dynamics of mitochondrial metabolism, the kinetics of the SCL25A51 carrier need to be further studied.

## 5. Mitochondrial NAD Transport and Protein Acetylation 

Enzyme activity is fine-tuned by post-transcriptional and post-translational modifications. The expression levels of mitochondrial proteins, including mitochondrial carriers that are encoded by nuclear DNA, are controlled by peroxisome proliferator-activated receptor gamma coactivator 1-alpha (PGC-1α), which is a master regulator of mitochondrial biogenesis [83,84]. Although mitochondrial carriers have been studied for the last 20 years, there is very little known about their regulation by post-translational modification. Similarly, it is not known whether the mammalian NAD carrier SLC25A51 activity can be regulated by post-translational modifications. Recently, it was reported that fasting can induce a higher expression of SCL25A51 [85]. Since fasting upregulates mitochondrial Sirt3, which uses intramitochondrial NAD, the required enhanced mitochondrial NAD uptake to sustain Sirt3 activity is in this way ensured. Sirt3 deacetylates several enzymes of the TCA cycle, respiratory chain, and ATP synthase [86,87,88]. Therefore, the NAD transport from the cytosol will have modulatory effects beyond influencing the rate of TCA-cycle metabolism, as it will also influence the rate of citrate generation, its transport into the cytosol, and downstream cytosolic acetyl-CoA generation by ATP-citrate lyases (ACLY), affecting histone acetylation, gene expression, and lipid metabolism. 

Perturbed NAD metabolism was observed under conditions of neurodegenerative diseases following acute brain injury, as well as during diabetic conditions or aging (for review, see [89]). Particularly, the recovery of substantially reduced mitochondrial NAD pools requires an intact and functional SCL25A51 carrier. The administration of NAD precursors (Nam, NMN, or NR) demonstrated a clear protective effect against several pathologies including ischemic and traumatic brain injury [3,71,90], neurodegenerative diseases, diabetic conditions, and aging processes. Thus, this suggests that the SCL25A51 carrier is functional under these pathologic conditions, allowing for the replenishment of mitochondrial NAD pools. This is because NAD is synthetized from the NMN, Nam, or NR in the cytosol and needs to be transported via SCL25A51 into the mitochondrial matrix. Consequently, a damaged or dysfunctional SCL25A51 carrier will have detrimental consequences for cellular bioenergetic metabolism and survival due to inhibited NAD transport across the inner mitochondrial membrane. 

## 6. Pathophysiology of Perturbed NAD Homeostasis 

Several lines of evidence suggest that NAD degradation and the associated bioenergetic failure of cellular metabolism is a major factor in the pathophysiology of neurodegenerative diseases. This is because NAD depletion leads to the inhibition of all NAD-dependent enzymes, causing the disruption of corresponding downstream metabolic pathways. The depletion of cellular NAD pools can be a result of either reduced NAD synthesis or increased NAD catabolism. It is generally believed that the overactivation of PARP1 and CD38 plays a significant role in mechanisms of pathophysiology in acute brain damage, neurodegenerative diseases or aging. As a consequence of uncontrolled PARP1 activation, intracellular NAD pools are depleted, which leads to mitochondrial dysfunction and bioenergetic failure followed by cell death [91,92]. The increase in PARP1 activity and the associated reduction in cellular NAD pools was also shown in Alzheimer’s and Parkinson’s disease models [93]. Similarly, CD38 is activated after ischemic insult and it contributes to the degradation of NAD pools [44]. CD38 also contributes significantly to the age-related decline in NAD levels [94]. 

Several treatment strategies have targeted the preservation or restoration of NAD levels. One approach was to administer inhibitors of NAD-consuming enzymes. Thus, inhibitors of PARP1 or CD38 were used to prevent the reduction in cellular NAD pools during pathologic conditions such as ischemia and traumatic brain injury, or in models of Alzheimer’s and Parkinson’s disease [95,96,97,98]. Although the inhibition of these enzymes showed neuroprotection, these approaches also lead to the suppression of PARP1 and CD38 physiological functions. An alternative means of restoring NAD levels is the administration of NAD precursors that can boost NAD synthesis. Nicotinamide was used to facilitate NAD generation via the salvage pathway following ischemic insult [71,99]. NAD synthesis can be directly stimulated by the administration of an NMN or NR [3,100]. Interestingly, in addition to the dramatic neuroprotective effect, the NMN also partially reduced PARP1 and CD38 NAD glycohydrolase activity [3,44]. Thus, NMN administration prevented the degradation of brain tissue NAD pools, and also reduced the excessive increase in poly-ADP-ribosylation [3]. Furthermore, the ischemia-induced changes in protein acetylation and the post-ischemic increase in free radical levels were reversed in animals treated with the NMN [101]. In rat and mouse models of Alzheimer’s disease both the NR and NMN improved neuronal cell health, memory, and cognitive function [102,103,104], and also showed neuroprotection in models of Parkinson’s disease [105] or ALS [106].

Mitochondria are dynamic organelles that can move, fragment, or fuse to meet the spatial-specific metabolic demands in cells. Interestingly, cellular and mitochondrial NAD levels can affect the mitochondrial fission and fusion process. Under pathologic conditions of acute brain injury or neurodegenerative disease, mitochondria show extensive fragmentation [103,107,108,109,110]. However, during recovery following transient global cerebral ischemia, the fragmented mitochondria in ischemia-resistant CA3 and dentate gyrus neurons undergo fusion and regain their pre-ischemic morphology [107]. However, in the more vulnerable CA1 neurons, the mitochondrial fragmentation was irreversible [107]. NMN administration shifts the mitochondrial dynamics towards fusion, and the mitochondria in CA1 neurons become less fragmented [101]. The reduced NAD levels compromise the activity of NAD-dependent deacetylases, sirtuins, causing increased acetylation of cellular proteins. Correspondingly, a low intramitochondrial NAD level leads to increased acetylation of TCA-cycle proteins, respiratory chain subunits, and the key enzyme of the mitochondrial antioxidant defense system, manganese superoxide dismutase (MnSOD) [88]. As a result of MnSOD hyperacetylation, mitochondrial MnSOD activity is inhibited and mitochondrial superoxide generation is increased [111]. By increasing the post-ischemic mitochondrial NAD levels, NMN administration reversed the MnSOD hyperacetylation, reducing free radical levels and mitochondrial fragmentation [101]. These data suggest that using an NAD precursor as a neuroprotectant against neurodegenerative brain damage is effective due to multifactorial effects where several mechanisms that contribute to brain damage are reserved [89].

## 7. Conclusions

Considering the essential role of NAD homeostasis in mitochondrial respiratory functions and the downstream effects via the NAD-dependent Sirt3 deacetylation activity and poly-ADP-ribosylation-dependent modulation of mitochondrial proteins, further studies of the NAD exchange mechanisms between different subcellular compartments are essential. These studies will have a significant impact on our understanding of cellular bioenergetic metabolism and may reveal novel regulatory pathways and therapeutic targets that might be related to mechanisms of neurological diseases. 

## Data Availability

No new data were created.
